# Traumatic atlanto-axial rotatory subluxation and dens fracture with subaxial SCIWORA of Brown-Sequard syndrome: A case report

**DOI:** 10.1097/MD.0000000000025588

**Published:** 2021-04-23

**Authors:** Sung-Kyu Kim, Dong-Gune Chang, Jong-Beom Park, Hyoung-Yeon Seo, Yun Hwan Kim

**Affiliations:** a Department of Orthopaedic Surgery, Chonnam National University Medical School and Hospital, Gwangju; b Department of Orthopaedic Surgery, Inje University Sanggye Paik Hospital, College of Medicine, Inje University; c Department of Orthopaedic Surgery, College of Medicine, The Catholic University of Korea, Seoul, Korea.

**Keywords:** atlanto-axial rotatory subluxation, Brown-Sequard syndrome, dens fracture, transverse atlantal ligament

## Abstract

**Rationale:**

A case of traumatic atlanto-axial rotatory subluxation (AARS), dens fracture, rupture of transverse atlantal ligament (TAL), and subaxial spinal cord injury without radiographic abnormality (SCIWORA) of Brown-Sequard syndrome has never been reported in a child.

**Patient concerns:**

A 7-year-old boy presented to hospital with torticollis, neck pain, and limited neck rotation after a seat-belt injury sustained during a car accident. Neurologic examination revealed right-side motor weakness and left-side sensory abnormality, known as Brown-Sequard syndrome.

**Diagnosis:**

Radiologic examinations revealed type II AARS (Fielding and Hawkins classification), increased atlanto-dental interval (ADI) of 4.5 mm due to a type 1B TAL rupture (Dickman classification), a displaced transverse dens fracture along with an ossiculum terminale, and an intramedullary hemorrhage on the right side of the spinal cord at C3–4.

**Interventions:**

The patient immediately received methylprednisolone, and his motor weakness and sensory abnormality gradually improved. At the same time, the patient underwent initial halter traction for 2 weeks, but he failed to achieve successful reduction and required manual reduction under general anesthesia.

**Outcomes:**

At the 7-month follow-up visit, radiologic examinations showed a corrected type II AARS that was well maintained and normalization of the ADI to 2 mm. The reduced transverse dens fracture was well maintained but still not united. All clinical symptoms were significantly improved, except the remaining motor weakness of the right upper extremity.

**Lessons:**

To the best of our knowledge, this is the first report of traumatic AARS, dens fracture, TAL rupture, and subaxial SCIWORA of Brown-Sequard syndrome in a child. Appropriate diagnosis and careful treatment strategy are required for successful management of complex cervical injuries in a child.

## 1 Introduction

Atlanto-axial rotatory subluxation (AARS), also called traumatic torticollis, is a common cervical spine injury in children.^[1,2]^ However, AARS associated with dens fracture in children is a rare injury, and until now, few cases have been reported in the English literature.^[3,4]^ Moreover, a case with 2 additional injuries, rupture of transverse atlantal ligament (TAL), and subaxial spinal cord injury without radiographic abnormality (SCIWORA) of Brown-Sequard syndrome^[5]^ to these 2 upper cervical spine injuries has never been reported in the English literature. Herein, we describe the detailed radiologic findings, treatment process, and result of an extremely rare and complex child cervical spine injury.^[1,6,7]^

## 2 Case report

**Figure 1:**
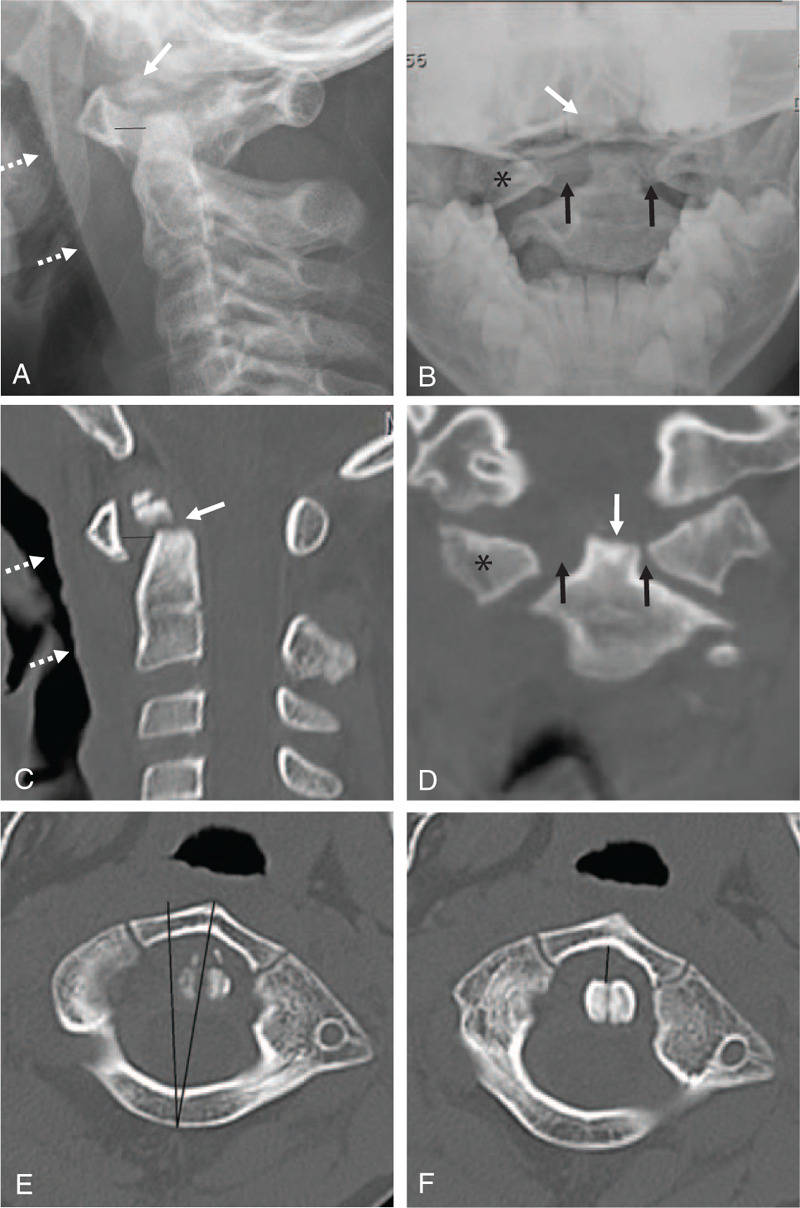
Lateral radiograph (A) showing anteriorly displaced transverse dens fracture along with ossiculum terminale (white arrow), increased atlanto-dental interval (ADI) of 4.5 mm (dark line), and increased anterior soft tissue swelling (dotted white arrows). Open mouth view (B) showing transverse dens fracture (white arrow), asymmetric distance between the lateral mass and dens (dark arrows), and anteriorly displaced right lateral mass of C1 (asterisk). Sagittal (C) and coronal (D) reconstructed computed tomography (CT) scans showing the same finding s more clearly. Axial CT (E and F) scans clearly showing anteriorly displaced and rotated right lateral mass of C1 to the left side and increased ADI of 4.5 mm, which indicating type II atlanto-axial rotatory subluxation (AARS) (according to Fielding and Hawkins classification).

**Figure 2:**
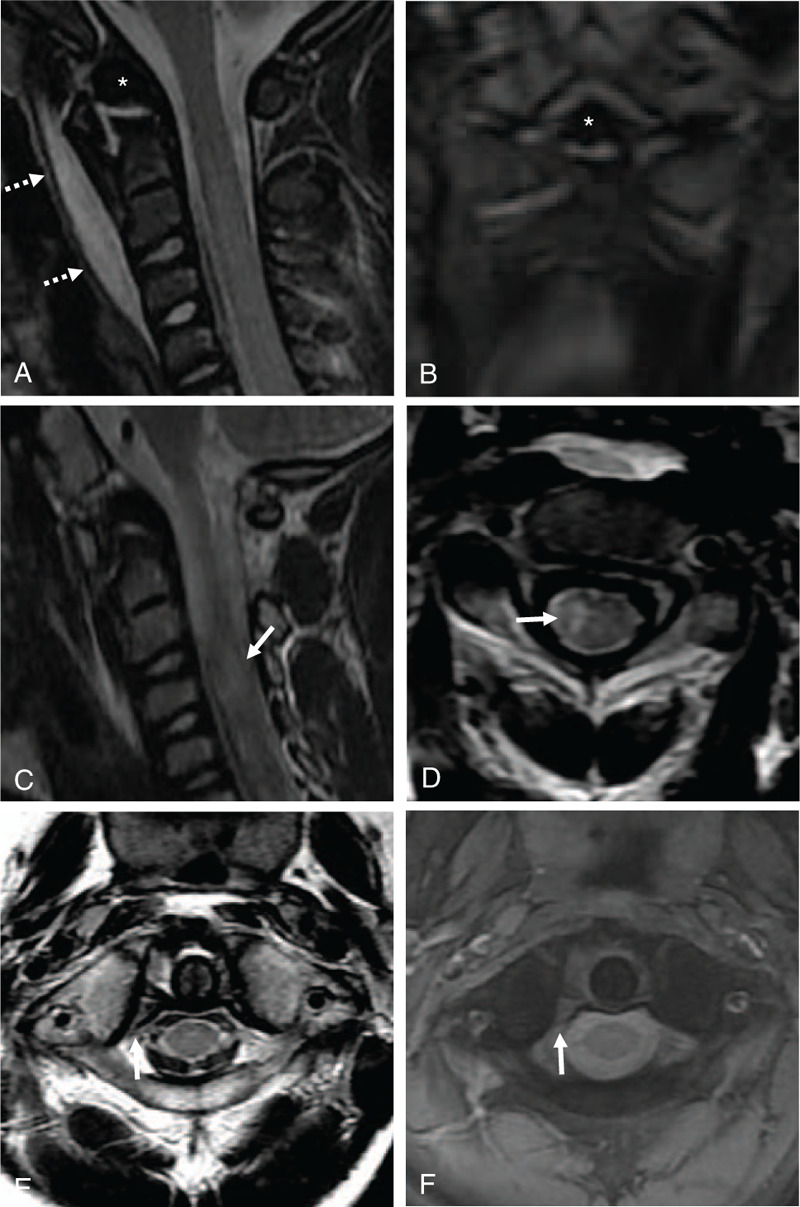
Midline sagittal and coronal (A and B) magnetic resonance imaging (MRI) showing retropharyngeal hematoma (dotted white arrows) and transverse dens fracture (white asterisks). Right parasagittal and axial T2-weighted (C and D) MRI showing intramedullary hemorrhage that occupied about 35% of the right side of C3–4 spinal cord (white arrows). Axial T2- and T1-weighted (E and F) MRI showing injury of transverse atlantal ligament (TAL) (white arrows) on right side of lateral mass of C1, which was type 1B TAL injury (according to Dickman classification).

A 7-year-old boy presented to our hospital complaining of torticollis, neck pain, and right-side hemiplegia that occurred after a seat-belt injury sustained during a car accident 1 day previous. Clinical examination showed tenderness and limited neck rotation. Neurological examination showed motor weakness on the right side (right upper extremity, muscle power grade 0/5; right lower extremity, 2–3/5; and left upper and lower extremities, 5/5) and no sensation of pain and temperature on the left side. Plain radiographs (Fig. 1A and B) revealed anteriorly displaced transverse dens fracture along with ossiculum terminale, increased atlanto-dental interval (ADI) of 4.5 mm, asymmetric distance between the lateral masses of C1, anteriorly displaced and rotated right lateral mass of C1 to the left side, and increased anterior soft tissue swelling that indicated a retropharyngeal hematoma. Sagittal and coronal reconstructed computed tomography (CT) scans (Fig. 1C and D) showed the same findings more clearly. Axial CT scans (Fig. 1E and F) revealed unilateral facet subluxation with increased ADI of 4.5 mm, indicating type II AARS. Sagittal and coronal magnetic resonance imaging (MRI) (Fig. 2A and B) revealed retropharyngeal hematoma and transverse dens fracture. Right parasagittal and axial MRI (Fig. 2C and D) revealed intramedullary hemorrhage that occupied about 35% of the right side of the C3–4 spinal cord. However, no bony injuries were identified at C3–4 or the subaxial spine. An evaluation and consideration of neurologic abnormalities and radiologic findings resulted in diagnosis of Brown-Sequard syndrome at C3–4. Axial MRI (Fig. 2E and F) revealed rupture of the TAL at the same right side of the C1 lateral mass, which was type 1B TAL injury (Dickman classification).^[6]^

**Figure 3:**
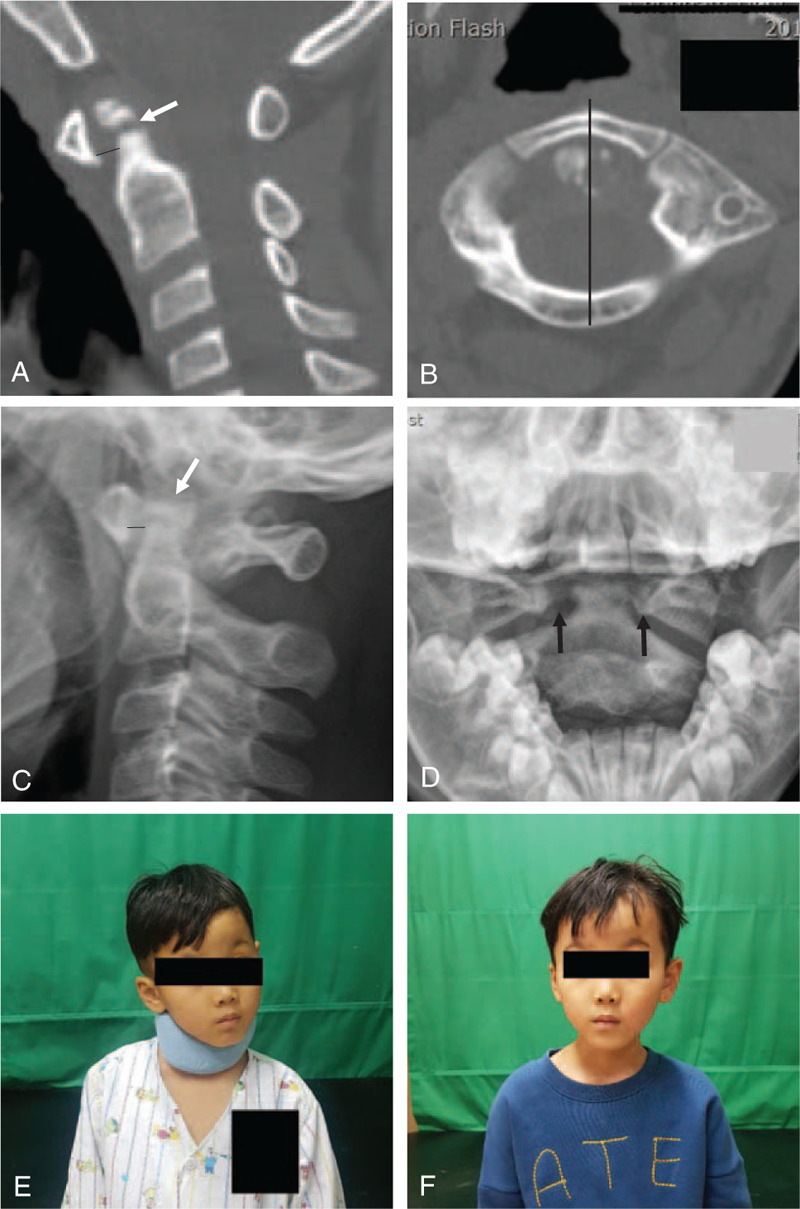
One month after closed reduction, follow-up sagittal reconstructed and axial (A and B) computed tomography (CT) scans clearly showed successful reduction of transverse dens fracture, decreased ADI of 3 mm (dark line), and correction of type II atlanto-axial rotatory subluxation (AARS). Seven months after closed reduction, follow-up lateral radiograph and open mouth view (C and D) showed a well maintained reduced transverse dens fracture with ossiculum terminale (white arrow), corrected type II AARS (dark arrows), and normalization of atlanto-dental interval (ADI) of 2 mm (dark line). However, transverse dens fracture was not still united. Compared to initial traumatic torticollis due to type II AARS (E), a follow-up clinical photo (F) showed normal neck posture with correction of torticollis.

On admission, the patient's body weight was 22 kg, and he received methylprednisolone for SCIWORA of Brown-Sequard syndrome at C3–4. An infusion of methylprednisolone 500 mg for 3 hours was repeated for 5 days according to recommendation from physicians of the departments of pediatrics and neurology. At the same time, continuous traction was applied using a halter with 1-kg of weight. The weight was gradually increased to 3-kg over a period of 2 weeks, but he failed to achieve successful reduction. Therefore, manual reduction was attempted with the patient under general anesthesia, and reduction was finally achieved. The patient wore a custom-made Minerva brace for 6 weeks. The motor weakness and sensory abnormality gradually improved after methylprednisolone treatment (right upper extremity, muscle power grade 3/5; right lower extremity, 4–5/5). One month after closed reduction, follow-up CT (Fig. 3A and B) scans showed successful reduction of anteriorly displaced transverse dens fracture along with ossiculum terminale, decreased ADI of 3 mm, and correction of type II AARS. Seven months after closed reduction, follow-up plain radiographs (Fig. 3C and D) revealed corrected type II AARS that was well maintained and normalization of ADI to 2 mm. The reduced transverse dens fracture along with an ossiculum terminale was well maintained but still not united. The motor grade was slightly improved to the right upper extremity 3–4/5 and the right lower extremity 4–5/5. Furthermore, pain and temperature sensation on the left side were returned to normal. Compared to his initial findings, the patient's torticollis and limited neck rotation were corrected with a normal neck posture (Fig. 3E and F). After attending another hospital for further rehabilitation, the patient was no longer followed.

## 3 Discussion

The overall incidence of pediatric spinal injuries in the United States has been reported to be 7.41/100,000.^[7–16]^ The cervical spine is the most common level, and the majority of the pediatric cervical spine injuries occur between the skull and the C4 vertebra.^[17–20]^ However, traumatic AARS associated with dens fracture in children is a rare and complex injury. To date, few cases have been reported in the English literature.^[3,4]^ Furthermore, there has been no report of a case involving additional TAL rupture and subaxial SCIWORA of Brown-Sequard syndrome along with these 2 upper cervical spine injuries with the following characteristics. First, the pediatric patient had type II AARS and anteriorly displaced transverse dens fracture simultaneously. The tip of the dens, ossiculum terminale, is not ossified at birth. Fusion of the ossiculum terminale to the rest of the dens occurs between 10 and 13 years of age.^[21]^ So, this physis is a vulnerable site for pediatric cervical spine injury at C1–2. Contrary to previous reports, dens fracture occurred transversely through the upper part of the dens, not the ossiculum terminale through the apical odontoid epiphysis.^[21,22]^ In addition, transverse dens fracture along with the intact ossiculum terminale were displaced anteriorly. These findings are very characteristic given that fractures in children commonly occur through the epiphysis or growth plate. Secondly, the aforementioned upper cervical spine injuries were accompanied by an additional type 1B TAL rupture as well as SCIWORA of Brown-Sequard Syndrome at C3–4. TAL rupture is essential for type II AARS that requires anterior displacement of 3-mm or greater.^[1]^ While neurologic deficits are infrequent in AARS, few studies have reported focal neurologic deficits or myelopathy due to marked AARS and severe canal compromise at C1–2.^[23,24]^ However, there have been no cases of associated Brown-Sequard syndrome in subaxial spine like our case other than C1–2.

The pediatric cervical spine injuries are different from those in adults due to the unique anatomy and relative immaturity of the pediatric cervical spine. The relatively larger head size observed in children results in greater flexion and extension injuries and more serious spinal injury.^[18,19]^Moreover, the highly flexible pediatric spine has greater tolerability to motion. The unique features observed in the pediatric population include a more elastic spinal column, greater ligamentous laxity, and immaturity of neck musculature, greater flexibility. Therefore, greater flexibility of the spinal column compared to the spinal cord can cause spinal cord injury (SCI). Furthermore, physiological wedging of immature vertebral bodies and horizontally-oriented facet joints also can cause greater incidence of dislocation or subluxation injuries.^[5]^ The fulcrum for maximum flexion shifts from the upper cervical spine to C3–4 at approximately age 6, and then it shifts to C5–6 in adolescence and early adulthood. Therefore, the upper cervical spine, especially C3–4, is more susceptible to flexion forces in children younger than 8 years, whereas the lower cervical spine is more commonly affected in children older than 9 years.^[20,21]^ The present patient was wearing a seat belt at the time of a car accident. Therefore, a relatively greater flexion-extension injury occurred, resulting in complex injuries of type II AARS, type 1B TAL rupture, and anterior displacement of transverse dens fracture. Because the patient was a 7-year-old boy, so C3–4 was the most vulnerable to flexion force, resulting in SCIWORA of Brown-Sequard syndrome at C3–4. Considering that C1–2 facet subluxation was right side, TAL rupture was right side, and intramedullary hemorrhage was right side, it is presumed that the flexion force acted asymmetrically, or the patient's neck posture was asymmetric at the time of the accident.

Previous studies have reported that MRI findings in pediatric patients with SCI may predict prognosis.^[19,20]^ While complete disruption of the cord and major hemorrhage more than 50% of the spinal cord are difficult to expect satisfactory neurologic recovery, minor hemorrhage less than 50% of the spinal cord is associated with a reasonable chance for partial recovery. The majority of pediatric patients with SCI is managed conservatively. The use of methylprednisolone to limit SCI and promote neurologic improvement is controversial.^[19,20]^ Moreover, since most studies were conducted in adult patients, application of methylprednisolone to pediatric patients is based on empirical experience, not scientific evidence. Administration of corticosteroids to pediatric SCI patients varies from 8% to 50% of the study subjects and is more controversial.^[20]^ In the current case, methylprednisolone was used according to the recommendations from a pediatrician and a neurologist because the intramedullary hemorrhage occupied about 35% of the C3–4 spinal cord, and neurologic deficits were severe. Fortunately, after use of methylprednisolone, the patient's motor weakness and sensory abnormality gradually recovered without complications. However, we believe that use of methylprednisolone in pediatric SCI patients should be carefully determined with full consideration of benefits and complications.

Type II AARS and dens fracture in children can be successfully treated by conservative measures, such as cervical collar, halter traction, and halo vest. Dickman et al classified TAL injuries into 2 types.^[6]^ Type 1 TAL injuries disrupt the ligament substance in its midportion (1A) or at its periosteal insertion (1B). Type 2 TAL injuries are fractures and avulsions involving the tubercle for insertion of the TAL on the C1 lateral mass. In addition, the authors also suggested that type 1 TAL injuries should be treated with early surgery, but type 2 TAL injuries should be treated conservatively. The TAL injury in the present case was type 1B, and, according to the recommendation by Dickman et al, should have been treated with surgical stabilization. However, our case included complex upper cervical spine injuries consisting of type II AARS, type 1B TAL rupture, and transverse dens fracture. So, we decided to treat the child patient conservatively first and achieved satisfactory outcomes for all 3 injuries. In the study of Dickman et al, they excluded TAL injury of children under the age of 14 years from the study. Greene et al also reported that dens fractures with TAL injuries should be considered for early surgical stabilization. However, Greene et al’ study also included all adult patients with type II dens fractures.^[25]^ Because the potential healing capacity of pediatric patients is very different from that of adult patients, we thought it unreasonable to apply the adult TAL treatment recommendations from Dickman et al and Greene et al to a pediatric patient with a TAL injury. Therefore, further investigation is needed to establish the appropriate treatment strategy specifically for pediatric TAL injury.

In conclusion, to the best of our knowledge, this is the first report of traumatic type II AARS, transverse dens fracture, type 1B TAL rupture, and subaxial SCIWORA of Brown-Sequard syndrome in a child. Appropriate diagnosis and careful treatment strategy are required for successful management of complex cervical injuries in a child.

## Author contributions

**Conceptualization:** Sung-Kyu Kim, Jong-Beom Park.

**Data curation:** Hyoung-Yeon Seo.

**Investigation:** Dong-Gune Chang, Jong-Beom Park, Hyoung-Yeon Seo.

**Methodology:** Yun Hwan Kim.

**Project administration:** Jong-Beom Park.

**Resources:** Hyoung-Yeon Seo.

**Supervision:** Dong-Gune Chang, Jong-Beom Park.

**Validation:** Hyoung-Yeon Seo.

**Visualization:** Yun Hwan Kim.

**Writing – original draft:** Sung-Kyu Kim.

**Writing – review & editing:** Dong-Gune Chang, Jong-Beom Park.
